# Geometric analysis of indications for minimally invasive distal metatarsal osteotomy in treatment of hallux valgus

**DOI:** 10.1186/s13018-015-0304-7

**Published:** 2015-10-17

**Authors:** Blaž Mavčič

**Affiliations:** University Medical Centre Ljubljana, Department of Orthopaedic Surgery, Zaloška 9, SI-1000 Ljubljana, Slovenia

**Keywords:** Minimally invasive distal metatarsal osteotomy, Geometric analysis, Hallux valgus

## Abstract

**Background:**

Minimally invasive distal metatarsal osteotomy (MIDMO) is to be indicated for all patients with angles of IMA <20° and HV <40°, but many authors doubt whether this procedure is capable of correcting all types of hallux valgus deformities. The aims of this study were to perform a geometric analysis of MIDMO indications and to show which preoperative radiological parameters are necessary to achieve sufficient contact between bone fragments and sufficient correction with this operative technique.

**Methods:**

A geometric mathematical model in AP and lateral radiographic plane was created based on preoperative measurements of the intermetatarsal angle (IMA), subcapital metatarsal width, medial bunion eminence, and metatarsal length. MIDMO was simulated with possible dorsal/plantar fragment displacement in order to assess postoperative contact between fragments (either 4–5 mm or half of the metatarsal width) and sufficient correction (postoperative IMA 8°).

**Results:**

The metatarsal neck should be at least 8 mm wider from the bunion eminence to achieve the minimally required contact between fragments. For sufficient correction, the metatarsal head translation should be at least 0.018 of the metatarsal length for every degree of IMA reduction. The medial bunion eminence, in comparison with metatarsal width/length size, determines whether MIDMO is a suitable procedure for a given patient.

**Conclusions:**

MIDMO cannot sufficiently correct all deformations within the boundaries of IMA angle <20° and HV angle <40°. In patients with large eminences and narrow metatarsals, complications related to insufficient postoperative fragment contact can be expected, while sufficient hallux valgus correction in patients with small eminences and long metatarsals is questionable.

**Electronic supplementary material:**

The online version of this article (doi:10.1186/s13018-015-0304-7) contains supplementary material, which is available to authorized users.

## Background

Minimally invasive distal metatarsal osteotomy (MIDMO) [1–6] is to be indicated for all patients with angles of IMA <20° and HV <40° [7–13], but many authors doubt whether this procedure is capable of correcting all types of hallux valgus deformities [14–17]. Minimally, invasive distal metatarsal osteotomy (MIDMO) for hallux valgus treatment was originally introduced by Bösch in 1990 [1, 2]; since then, several other authors have published their own modifications of the original technique [3–7]. The common features of above techniques include subcapital osteotomy of the first metatarsal, lateral translation of the head, and blocking the head with a K-wire inserted proximally into the medullary canal of the first metatarsal. Many surgeons continue to use the MIDMO method, and studies from various independent sources report good to excellent results [8–13] due to the small incision required, as well as less postoperative pain and wound healing problems [5]. However, contrary reports tend to emphasize that MIDMO is not capable of correcting all types of hallux valgus deformities [14]. No randomized studies in this field have been reported [15]. Past studies underreported rates of complications (malunion, nonunion, and osteonecrosis) and recurrence, and the overall cost-effectiveness is unclear [16, 17].

Both authors who report good clinical results and the authors who criticize the use of the MIDMO have thus far been using unchanged indications of the procedure as outlined by the original authors (IMA angle <20° and HV angle <40°) [3–6]. Geometric analyses have been done for different types of distal metatarsal osteotomies [18–22], but these analyses were not specific for MIDMO with perpendicular subcapital osteotomy, did not take into account the metatarsal length and dorsal/posterior displacement of the distal fragment [21], or were too complex to be used on larger numbers of patients in the clinical setting without 3D imaging [22]. MIDMO significantly differs from other distal osteotomy techniques, because it retains the medial bunion eminence, and the amount of contact and correction is invariably defined by the operative technique itself. Since lateral translation is driven by the K-wire insertion into the first metatarsal canal, the operative technique enables only one possible magnitude of the metatarsal head lateral translation with slight variations in the osteotomy inclination and dorsal or posterior displacement of the distal fragment [3–6]. Thus, the geometric analyses published thus far are not suitable to answer the two important questions before the surgeon even considers using MIDMO in a given patient: (1) Will the given metatarsal head lateral translation with this technique result in sufficient contact between osteotomy fragments? (2) Will the given metatarsal head lateral translation result in sufficient hallux valgus correction with this technique? These two questions cannot be answered with the criteria of “IMA angle <20° and HV angle <40°” [1–6] alone; thus far, there have there been no other specific epidemiological studies published to show which patients will benefit from MIDMO.

The aims of this study were to perform a geometric analysis of the indications for MIDMO in the treatment of hallux valgus and, thereby, to show which preoperative radiographic parameters are necessary to achieve sufficient contact between fragments and sufficient correction with this operative technique.

## Methods

### Geometric analysis of preoperative measurements

**Fig. 1:**
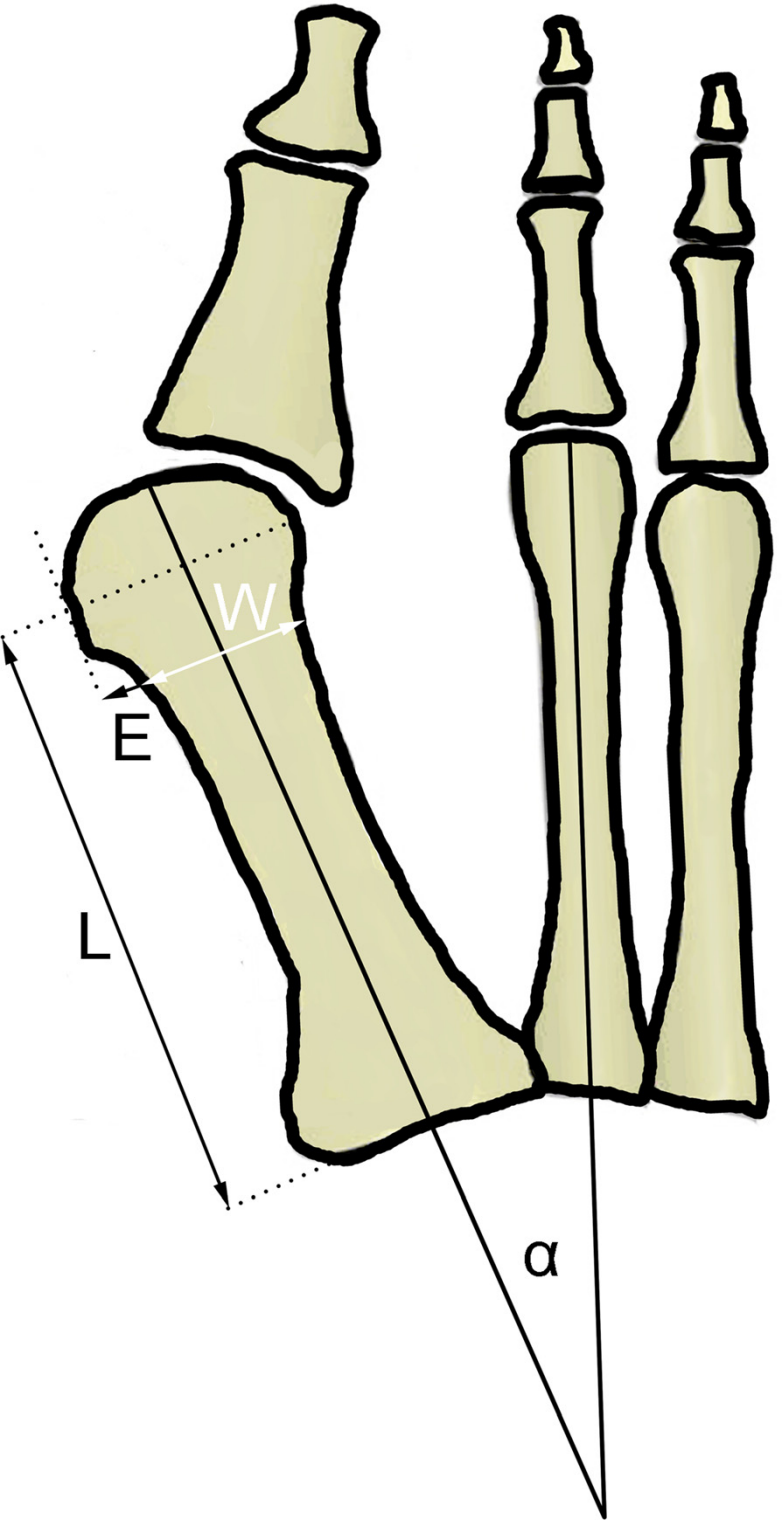
Geometrical model of hallux valgus in the anterior-posterior plane. The model is based on four radiographic parameters: *α* the preoperative intermetatarsal angle (first and second metatarsal) [degrees], *W* subcapital width of the first metatarsal [millimeters], *E* eminence-bunion protrusion from the metatarsal bone shaft [millimeters], *L* length of the first metatarsal from its base to the head center [millimeters]

In order to analyze status of the first metatarsal bone before and after minimally invasive distal metatarsal osteotomy (MIDMO), a mathematical geometric model has been created representing the first and the second metatarsal of the right foot in the anterior-posterior plane (Fig. 1). The entire model is based on four radiographic parameters that need to be measured from the anterior-posterior foot radiograph in the standing position:

α: the preoperative intermetatarsal angle (first and second metatarsal) [degrees]
W: subcapital width of the first metatarsal [millimeters]
E: eminence-bunion protrusion from the metatarsal bone shaft [millimeters]
L: length of the first metatarsal from its base to the head center [millimeters]

### Mathematical simulation of MIDMO

**Fig. 2:**
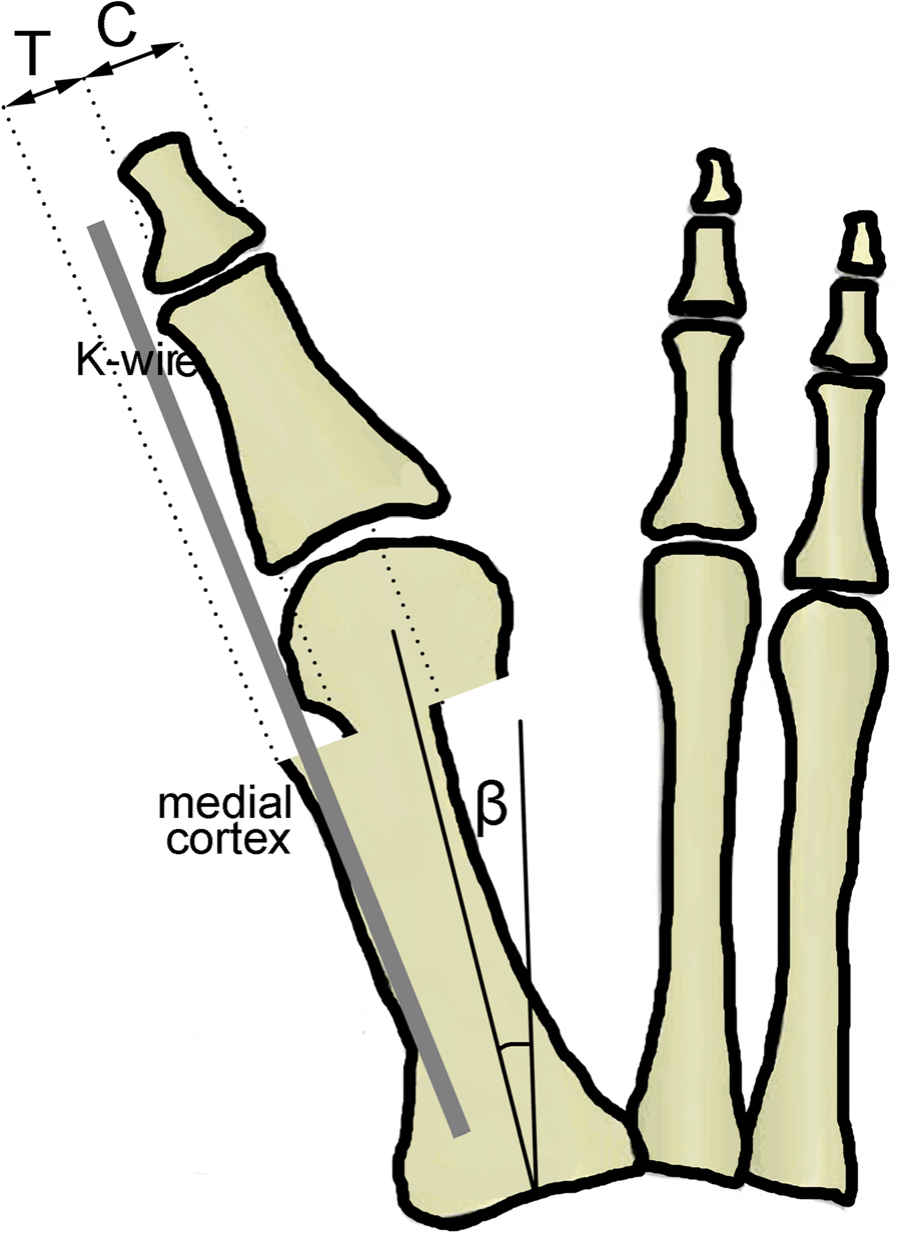
Lateral translation of the metatarsal head with the K-wire. Effects of MIDMO can be described with the following radiographic parameters: *T* lateral translation of the metatarsal head in the osteotomy plane [millimeters], *C* contact between fragments after osteotomy and displacement [millimeters], *β* the postoperative intermetatarsal angle (first and second metatarsal) [degrees]

The main consequence of MIDMO is lateral translation of the metatarsal head in the osteotomy plane (Fig. 2). To represent the changes that occur after osteotomy, four additional geometric parameters are introduced into the presented mathematical model as shown in Fig. 2 and Fig. 4 (the lateral view is also necessary in order to assess the need for additional dorsal or plantar displacement of the distal osteotomy fragment):

x: additional dorsal or plantar displacement of the distal fragment [millimeters]
T: lateral translation of the metatarsal head in the osteotomy plane [millimeters]
C: contact between fragments after osteotomy and displacement [millimeters]
β: the postoperative intermetatarsal angle (first and second metatarsal) [degrees]

MIDMO can theoretically be performed at varying angles with regard to the metatarsal shaft [21]; however, in accordance with original recommendations [1–3], the osteotomy plane should be perpendicular to the longitudinal axis of the first metatarsal shaft (Fig. 2). After the osteotomy is performed, a 2.0 mm K-wire is inserted in the soft tissues adjacent to the distal and proximal phalanx and then further on into the first metatarsal medullary canal in order to push the metatarsal head laterally by pressing against the bunion eminence. Given that the mean thickness of the medial cortex in the first metatarsal is 1.5 mm [23], the magnitude of translation in the lateral direction with regard to the first metatarsal shaft then equals (Fig. 2)

1 $$ \begin{array}{l}T = E + 2.0\kern0.5em \mathrm{mm} + 1.5\kern0.5em \mathrm{mm} = E + 3.5\kern0.5em \mathrm{mm}\\ {}\kern1.62em \mathrm{K}\hbox{-} \mathrm{wire}\ \mathrm{width}\kern1.81em \mathrm{cortex}\ \mathrm{width}\end{array} $$

The contact between fragments after osteotomy and lateral displacement-translation *T* will equal (Fig. 2 and Fig. 4)

2 $$ C = W - T = W - E - 3.5\ \mathrm{mm} $$

**Fig. 3:**
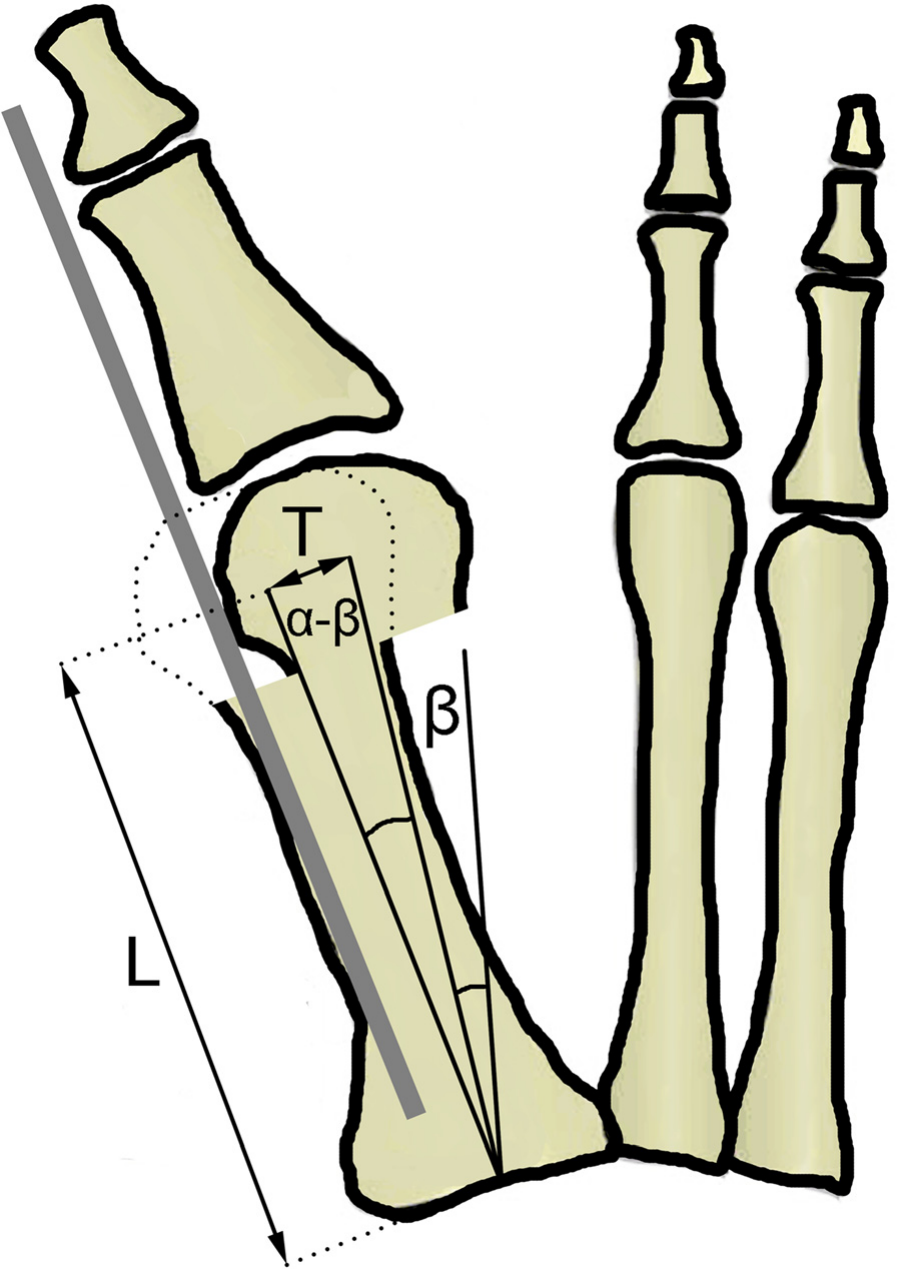
Intermetatarsal angle before/after lateral translation of the metatarsal head. With lateral translation of the metatarsal head the initial intermetatarsal angle *α* is reduced to *β*. The angle of correction (*α* − *β*) is part of the right triangle with adjacent side *L* and the opposite side *T*

The lateral translation of the metatarsal head center by the magnitude *T* results in the changed position of the reference points for the intermetatarsal angle measurement from preoperative *α* to postoperative *β* as follows (Fig. 3):

3 $$ T/L = \tan\ \left(\alpha -\beta \right) $$

Therefore, the postoperative intermetatarsal angle *β* can be computed as

4 $$ \beta \kern0.5em =\kern0.5em \alpha -\mathrm{atan}\kern0.5em \left(T/L\right) $$

The stability of the K-wire fixation in the relatively large medullary canal should be achieved by the strong forces of the metatarsal head fragment and soft tissues from the lateral side preventing any shift medially [1–6]. The intramedullary K-wire is usually removed 6 weeks after operation; in the mean time, patients are instructed to walk on their heel and wear postoperative shoes with flat rigid soles [4]. Due to problems with achieving distal fragment stability, some authors recommend adding a second K-wire proximally [4] or in some cases even a small dorsal locking-plate can be used. It should be noted, however, that any supplemental fixation methods do not affect the medial-lateral position of the metatarsal head, because it is determined entirely by the lateral push of the intramedullary K-wire.

## Results and discussion

### Contact between fragments after osteotomy

If the required minimal contact is expressed in terms of an absolute number 4–5 mm (for example 4.5 mm) [24], then Eq. 2 can be rewritten as

5 $$ 4.5\ \mathrm{mm} = W - E - 3.5\ \mathrm{mm} $$

6 $$ W = E + 8\ \mathrm{mm} $$

If the required minimal contact is expressed in terms of half metatarsal width [24], then Eq. 2 can be rewritten as

7 $$ 1/2W=W-E-3.5\ \mathrm{mm} $$

Therefore, it can be computed that the contact magnitude of the half metatarsal width will only be achieved if

8 $$ W = 2\ E + 7\ \mathrm{mm} $$

### Contact between fragments with displacement in the dorsal or plantar direction

**Fig. 4:**
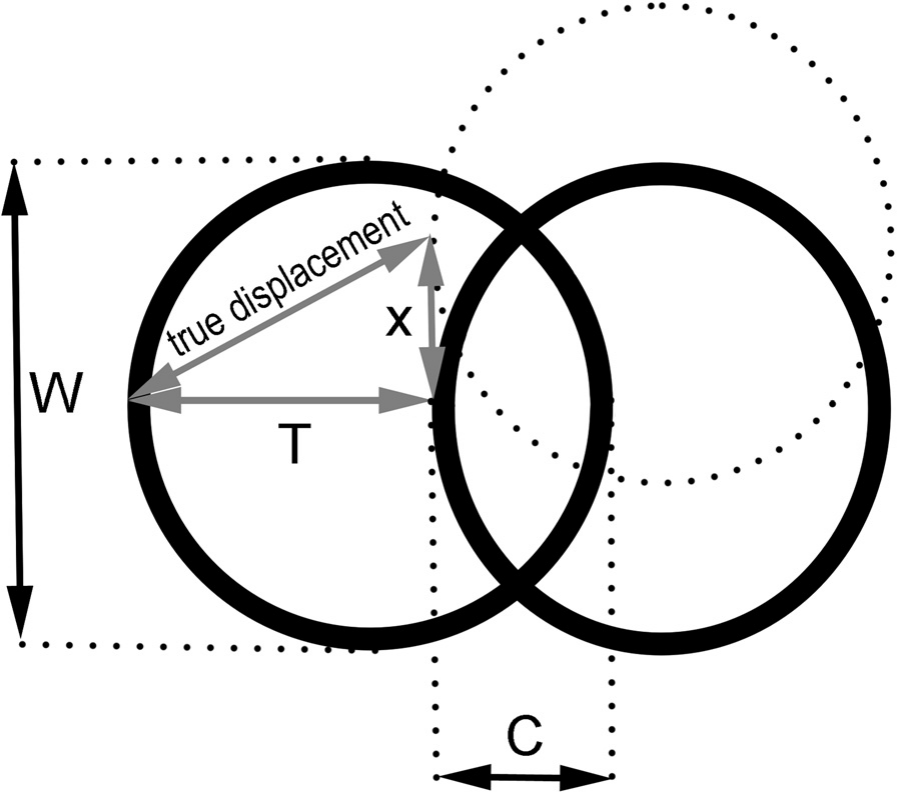
Additional dorsal or plantar displacement of the distal osteotomy fragment. When distal osteotomy fragment is displaced laterally by magnitude *T*, we get two approximately cylindrical fragments of diameter *W* with the distance between the edges of these cylinders being exactly *T*. If the distal osteotomy fragment is additionally displaced in the dorsal or plantar direction by the magnitude of *x* (i.e., in the lateral radiographic projection), the bony fragment overlap in the AP radiographic view remains apparently equal while the true displacement between the two cylinder edges is increased

When the distal osteotomy fragment is displaced laterally by magnitude *T*, two approximately cylindrical fragments of diameter *W* with the distance between the edges of these cylinders being exactly *T* are obtained (Fig. 4). Thus, the magnitude of contact between the two fragments equals *C* = *W* – *T*, as presented on Fig. 2 and Fig. 4 and previously outlined in Eq. 2.

However, if the distal osteotomy fragment is additionally displaced in the dorsal or plantar direction by a magnitude of *x* (i.e., in the lateral radiographic projection), the bony fragment overlap in the AP radiographic view remains apparently equal while the true distance between the two cylindrical edges is increased, and the true contact magnitude between the two fragments is thus reduced (Fig. 4). Note that the magnitude of hallux valgus correction in the AP radiographic plane remains unchanged regardless of the magnitude of *x*.

According to the Pythagorean theorem, on the three sides of a right triangle (*a*^2^ + *b*^2^ = *c*^2^), the true displacement between edges of cylindrical fragments in the case of additional dorsal or plantar displacement is (Fig. 4)

9 $$ \mathrm{true}\ \mathrm{displacement} = \sqrt{T^2 + {x}^2} $$

Thus, the true contact between fragments after osteotomy with lateral displacement *T* and additional dorsal or plantar displacement x will equal

10 $$ \mathrm{true}\ \mathrm{contact}=W-\sqrt{T^2+{x}^2} $$

If there is no additional dorsal or plantar displacement of the distal osteotomy fragment (i.e., if *x* = 0), Eq. 10 then becomes identical to Eq. 2.

### Achievement of sufficient hallux valgus correction after osteotomy

Most guidelines on the magnitude of hallux valgus correction recommend slight overcorrection with the final intermetatarsal angle value of 8° [1–6, 24]. Changes in the metatarsal angle after achieving the desired correction (Fig. 3) can, therefore, be described by using the value *β* = 8° in Eq. 3:

11 $$ T\ /\ L = tan\ \left(\alpha - 8{}^{\circ}\right) $$

When *T* is substituted with the expression from Eq. 2, the result is

12 $$ E = L \times tan\ \left(\alpha - 8{}^{\circ}\right) - 3.5\ \mathrm{mm} $$

Therefore, different preoperative values of the intermetatarsal angle *α* require different protrusion magnitudes of the bunion eminence in comparison to the metatarsal length in order to achieve sufficient correction to 8° (Table 1).

**Table 1:** Values of Eq. 12 for different physiological intermetatarsal angles *α*

Intermetatarsal angle *α* (°)	Intended correction (*α−*8°) (°)	Equation 12
12	4	*E* = 0.07 × L − 3.5 mm
14	6	E = 0.11 × L − 3.5 mm
16	8	*E* = 0.14 × L − 3.5 mm
18	10	*E* = 0.18 × L − 3.5 mm
20	12	*E* = 0.21 × L − 3.5 mm

For practical clinical use, Eq. 1 combined with values of Eq. 12 in Table 1 can be simplified to show that each additional degree of the intended hallux valgus correction requires additional lateral translation of the metatarsal head by 0.018 of the metatarsal length *L*:

13 $$ T \approx 0.018 \times L \times \left(\alpha - 8{}^{\circ}\right) $$

whereby (*α* − 8°) is regarded as numeric integer value, representing the number of degrees of intended correction.

### Preoperative planning for a given patient

**Fig. 5:**
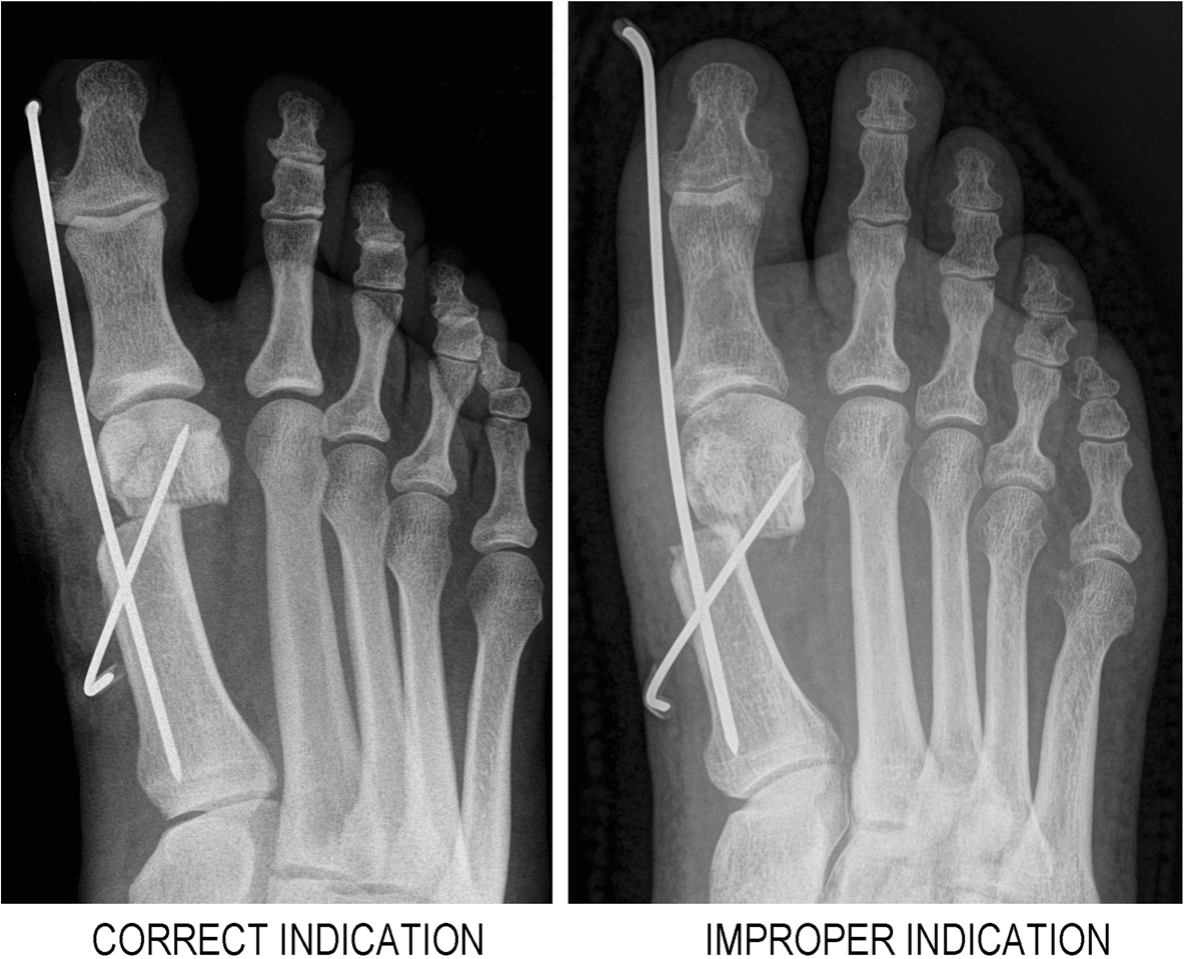
Clinical examples of correct and improper MIDMO indication. *Left* part of the figure shows correct indication for MIDMO with sufficient postoperative contact between fragments and optimal postoperative correction. *Right* part of the figure shows MIDMO with improper indication because of too large bunion exostosis (5.5 mm) for the given subcapital neck width (13 mm). Geometrical analysis of preoperative radiograph predicts only 4 mm of postoperative contact, and this was even further reduced to 3 mm due to intraoperative plantar displacement of the distal fragment, finally resulting in pseudoarthrosis

For a particular patient who has had the four basic radiographic parameters (*α*, *W*, *E*, *L*) measured from the anterior-posterior foot radiograph in the standing position and where possible additional dorsal or plantar displacement of the distal fragment (*x*) is taken into account, the expected magnitude of lateral translation (*T* or true displacement) and contact between osteotomy fragments (*C* or true contact) and the achievable correction (in terms of the expected postoperative intermetatarsal angle *β*) can easily be computed with the attached software tool (.XLS file). In order to achieve the minimally required contact between fragments, Eq. 6 shows that metatarsal neck in a given patient should be at least 8 mm wider from the bunion eminence (see examples of correct and improper MIDMO indication in Fig. 5). In addition, sufficient correction will only be achieved if the metatarsal head translation amounts to at least 0.018 of the metatarsal length for every degree of the desired IMA reduction, as presented in Eq. 13. Therefore, the size of the medial bunion eminence in comparison with the metatarsal width/length determines whether MIDMO is a suitable procedure for a given patient.

### Application of the presented methodology with epidemiological data

The findings of our study concur with the authors who have indicated that the planning of other distal metatarsal osteotomies [24] should also involve the width of the first metatarsal [18–22]. Since most distal metatarsal osteotomies include the removal of the medial bunion eminence, the size of this eminence and metatarsal length have not thus far been considered to be an important factor of the hallux valgus pathology [18–22, 25]. Unlike previous geometric analyses, the presented study clearly shows that the medial protrusion of the bunion eminence is one of the crucial parameters of preoperative planning for the MIDMO operative technique.

Previously published epidemiological data indicate which patients are more or less suitable for MIDMO. If the estimated average size of the medial bunion eminence in patients with hallux valgus *E* = 4.5 mm [24, 25] is inserted into Eq. 6, the minimal required subcapital width of the first metatarsal will be 12.5 mm, which already exceeds the average female metatarsal size [19]. Furthermore, if the surgeon wants to have a contact surface of at least half of the metatarsal width (*E* = 4.5 mm inserted into Eq. 8), the minimal required subcapital width is computed as 16 mm, i.e., the size only found in large male feet [19]. With regard to achieving sufficient hallux valgus correction (Table 1), the average sized medial bunion eminence *E* = 4.5 mm [24, 25] could sufficiently correct the intermetatarsal angle of 16° only in metatarsals with the length (base ↔ head center) of 57 mm and the intermetatarsal angle of 20° only in metatarsals with the length (base ↔ head center) of 38 mm, which is far below the mean metatarsal length of adults, particularly males [26]. The epidemiological data, therefore, shows that MIDMO cannot sufficiently correct all deformations within the boundaries of IMA angle <20° and HV angle <40°.

Limitations of the presented mathematical model include its two-dimensionality, the lack of incorporation of soft tissues and joint (in)congruency into the model and inability to analyze the impact of the hallux valgus angle on correction. The issue of two-dimensionality has been improved by including analysis of the dorsal/plantar fragment displacement so that the geometric model takes into account translational changes both in AP and lateral radiographic planes. With regard to soft tissues and joint congruency, it should be noted that the original technique of MIDMO does not consider any soft tissue corrections or releases to be necessary [1–6]. Therefore, the presented mathematical model can be considered adequate for the study of the particular MIDMO operative technique, although it would not be suitable for analyses of other distal metatarsal osteotomies requiring soft tissue release.

## Conclusions

Minimally invasive distal metatarsal osteotomy (MIDMO) cannot sufficiently correct all deformations within the boundaries of IMA angle <20° and HV angle <40°. The presented study quantitatively shows how the size of the medial bunion eminence determines whether MIDMO is suitable for a given patient: in patients with large eminences and narrow metatarsals, complications related to insufficient postoperative contact between fragments (pseudoarthrosis, fragment displacement) can be expected, while in patients with small eminences and long metatarsals, the expected complications would be related to insufficient hallux valgus correction (persistent pain, early recurrence). The presented geometric analysis can be a useful preoperative planning tool to aid in deciding which patients will benefit most from MIDMO and to assess the possible causes of failed surgery.

### Availability of supporting data

All computations described within this paper can be performed with the attached freeware computer program (.XLS file) Additional file 1.

## Additional file

Additional file 1:
**Freeware computer program - MIDMO.** A freeware computer program in .XLS format allows preoperative planning of MIDMO in individual patients. The required input parameters include measurements of α, W, E, L and x from anetrior-posterior standing radiograph of the foot. (XLS 27 kb)

## Abbreviations

C: Contact between fragments after osteotomy and displacement [millimeters]
E: Eminence-bunion protrusion from the metatarsal bone shaft [millimeters]
L: Length of the first metatarsal from its base to the head center [millimeters]
MIDMO: Minimally invasive distal metatarsal osteotomy
T: Lateral translation of the metatarsal head in the osteotomy plane [millimeters]
W: Subcapital width of the first metatarsal [millimeters]
x: Additional dorsal or plantar displacement of the distal fragment [millimeters]
α: The preoperative intermetatarsal angle (first and second metatarsal) [degrees]
β: The postoperative intermetatarsal angle (first and second metatarsal) [degrees]
